# Fabrication and Multiscale Structural Properties of Interconnected Porous Biomaterial for Tissue Engineering by Freeze Isostatic Pressure (FIP)

**DOI:** 10.3390/jfb9030051

**Published:** 2018-08-24

**Authors:** Mythili Prakasam, Ali Chirazi, Grzegorz Pyka, Anna Prokhodtseva, Daniel Lichau, Alain Largeteau

**Affiliations:** 1 CNRS, Univ. Bordeaux, ICMCB, UMR 5026, F-33600 Pessac, France; alain.largeteau@u-bordeaux.fr; 2 Thermo Fisher Scientific, Impasse Rudolf Diesel, 33700 Merignac, France; Ali.Chirazi@fei.com (A.C.); DanielLichau@fei.com (D.L.); 3 Thermo Fisher Scientific, Vlastimila Pecha 1282/12, 62700 Brno, Czech Republic; Grzegorzpyka@fei.com (G.P.); Anna.Prokhodtseva@fei.com (A.P.)

**Keywords:** tissue engineering, porous materials, microstructure, biomaterials, bone regeneration

## Abstract

Biomaterial for tissue engineering is a topic of huge progress with a recent surge in fabrication and characterization advances. Biomaterials for tissue engineering applications or as scaffolds depend on various parameters such as fabrication technology, porosity, pore size, mechanical strength, and surface available for cell attachment. To serve the function of the scaffold, the porous biomaterial should have enough mechanical strength to aid in tissue engineering. With a new manufacturing technology, we have obtained high strength materials by optimizing a few processing parameters such as pressure, temperature, and dwell time, yielding the monolith with porosity in the range of 80%–93%. The three-dimensional interconnectivity of the porous media through scales for the newly manufactured biomaterial has been investigated using newly developed 3D correlative and multi-modal imaging techniques. Multiscale X-ray tomography, FIB-SEM Slice & View stacking, and high-resolution STEM-EDS electronic tomography observations have been combined allowing quantification of morphological and geometrical spatial distributions of the multiscale porous network through length scales spanning from tens of microns to less than a nanometer. The spatial distribution of the wall thickness has also been investigated and its possible relationship with pore connectivity and size distribution has been studied.

## 1. Introduction

Repairing of human injured tissues with the help of biomaterials was evidenced even in the pre-historic period [1]. Usage of metals such as gold for dental applications was observed even in the Roman era [2]. At present, huge research progress is observed in the case of bone tissue engineering. Bone tissue engineering is a field with continuous evolution and the demand of this field is continuously increasing owing to the growing population. In addition, bone tissue engineering is also in demand due to orthopedic reconstructions required in the case of trauma, birth defects, and degeneration. Various types of implants are employed for orthopedic applications such as autografts, allografts, and xenografts. Autografts have the upper hand in terms of superior bone fusion whereas allografts and xenografts have high probability in transmitting pathogens and causing inflammatory reactions in the human body. The aforesaid issues have led to the development of synthetic biomaterials for musculoskeletal tissue engineering to satisfy the requirements of orthopedics.

Human bone can be defined as a composite of hydroxyapatite [1] (HAp) Ca_10_ (OH)_2_(PO_4_)_6_, type-I collagen, water, cells, and lipids. Bones [2,3,4,5,6,7,8,9,10] are formed in the body as a result of the osteoblast matrix formed by HAp crystals. Bone is comprised of two distinct forms: one is porous (cancellous bone) and the other dense (cortical bone). Cancellous bone contains hemocytoblasts, proerythroblasts, and bone marrow. Cancellous bone has a lower Young’s modulus and is more elastic compared to cortical bone. The porous structure consists of pore sizes in the range of 200–500 μm, and cancellous bone constitutes 30%–90% of the porosity. The porosity content alters depending on the load, age, and health state of the bone. The constituents of human bone are 60%–70% bone mineral (Ca_10_ (PO_4_)_6_(OH)_2_), 10%–20% collagen, and 10%–20% water. In the 1920s, stainless steel metallic implants were developed for bone repair. Later, artificial bone implants were based on ceramics, composites, metals, and polymers for bone engineering [3,4,5,6].

Synthetic scaffolds developed should be capable of withstanding the mechanical loading, structural integrity, and possibility to support or host tissue and allow cell proliferation. Development of cancellous bone implants primarily depends on the structural design of the scaffold such as porosity, pore shape and size, interconnectivity of pores, orientation, and control of microstructure. Well defined mechanical modulus and porosity of the scaffold will help in accelerating the bone tissue regeneration and support growth of new bone tissue cells. Mechanical properties of natural bone are found to differ based on the asymmetry and anisotropy. Further, the highest modulus is observed in the bone when the loading axis is perpendicular to the axis of the long bone [7]. Research data has shown an estimation of minimum pore size of ~50 µm for blood vessel formation and a ~200 µm for osteonal in-growth [8]. Both porosity and their architecture are critical in gauging the fluid transport rate through porous bioceramics, which determines the rate and the degree of the bone growth in vivo [9].

The fabrication of porous scaffolds with complex architectures represents a challenge in tissue engineering. Recent studies have shown that it is possible to construct tissue-engineered bone repair scaffolds with tight pore size distributions and controlled geometries using rapid prototyping techniques. Despite the wide use of porous scaffolds, its design, geometry optimization, and mechanical assessment require further developments and studies for successful integration in tissue engineering. Calcium phosphate scaffolds are regarded as an interesting material for scaffold application. Calcium phosphate (CP) based materials aid in osteoblast adhesion and proliferation [10,11]. Various processing routes to fabricate CP-compounds include uniaxial compaction, cold or hot isostatic pressing, granulation, loose packing, slip casting, gel casting, pressure mold forming, injection molding, polymer replication, extrusion, slurry dipping, and spraying. In addition, formation of ceramic sheets by tape casting is also widely employed. Sintering of bioceramics is also well-known [12]. 

Porous biomaterials consolidated at low temperature with increased mechanical strength can enable inclusion of therapeutic molecules and is one of the domains of investigation at ICMCB-CNRS (Institut de Chimie de la Matière Condensée de Bordeaux-CNRS). The present article discusses the innovative experimental methodology used in the fabrication of porous scaffolds by freeze isostatic pressing (FIP). To demonstrate the feasibility of FIP, TiO_2_ anatase phase was selected. TiO_2_ has various applications such as in dye-sensitized solar cells, hydrogen production and storage, sensors, rechargeable batteries, electro catalysis, self-cleaning and antibacterial surfaces, and photocatalytic cancer treatment. No influence of the fabrication technology on the chemical composition of the starting compounds should be altered. To show the proof of concept on the non-influence of the fabrication technology on the composition, we have used TiO_2_ in the present work. The interconnected porosity and the pore volume were studied by employing 3D-correlative and multimodal imaging techniques to give an insight about the pore structure characteristics, homogeneity, and interconnectivity. This type of an integrated approach will throw light on pore size range, their size and form by employing a wide variety of imaging techniques such as multiscale X-ray tomography, FIB-SEM Slice & View stacking, and high-resolution STEM-EDS electronic tomography. This combined approach allows quantification of morphological and geometrical spatial distributions of the multiscale porous network through length scales spanning from tens of microns to less than a nanometer. The spatial distribution of the wall thickness was investigated as well and its possible relationship with pore connectivity and size distribution were studied and is discussed in detail in this article. The observations of the microstructure through these imaging techniques enabled us to quantify and validate the efficiency of our fabrication technique for porous biomaterials used in bone tissue engineering applications.

## 2. Results

Simultaneous study of the nano- and micro structural properties of materials and quantitative assessment of their relationship with the micro- or macroscopic functional properties of the materials are crucial prerequisites for development of imaging for porous biomaterials. The 3D imaging methodology will also assist in quantitative and qualitative analyses when used in conjunction with other experiments requiring nanotoxicity and cell interaction studies. Scaffolds, being an integral part of bone tissue engineering, require their three-dimensional area to be quantified. Three-dimensional information on scaffolds will help to analyze properties such as mechanical support, protein production through biochemical and mechanical interactions to aid in cell attachment, assisting in bone tissue formation. Chemical composition and fabrication methodology are the major parameters to judge their suitability to be used as scaffold. Fabrication methodology governs the pore size, pore volume, and mechanical strength of the scaffold and their performance. To date, conventionally used techniques for fabrication of porous scaffolds involve foaming, solvent casting, leaching, freeze casting, thermally induced phase separation, and additive manufacturing techniques. Apart from pore size and pore volume, the interconnectivity of the pores is essential to judge their role in their functioning. Open and interconnected pores allow nutrients and molecules into the inner part of the scaffold to enable vascularization. High porosity presents high surface area per unit volume. Biodegradation process is highly dependent on pore parameters and cell-mediated process through chemical dissolution to replace the scaffold with new bone. Pore sizes >300 µm of scaffolds assists in vascularization and enhanced bone formation. Reports on poly—(d, l-Lactic acid) scaffolds with pore sizes in the range of 320–420 µm proved to produce a well-organized collagen network. However, when the pore sizes were smaller, <270 µm prevented osteosarcoma derived osteoblasts to proliferate and produce extracellular matrix. High pore volume also aids in compressive strength. 

The scaffold fabricated should initially support and resist the host tissue for optimum bone healing. The degradation and mechanical properties depend on the pore size, geometry, and load application direction. The pore size and morphology are tailored and controlled based on the fabrication methodologies. Interconnectivity of the scaffolds is also essential to analyze but is difficult to control and analyze during fabrication methodology. Additive manufacturing and rapid prototyping allows complex scaffolds shapes. Interconnectivity in porous resorbable bioceramics are of utmost importance in comparison to their pore and interconnectivity density. Microstructural studies have enabled the quantifying and qualifying of the scaffolds for tissue engineering. µ-CT scanning is used to quantify the pore size and porosity of the scaffolds. µ-CT imaging and analysis is used to determine porosity and pore sizes in 3D biomaterial scaffolds. To identify the interconnectivity further, microstructural analyses and software were developed.

## 3. Discussion

### Freeze Isostatic Pressure: FIP

Application of isostatic pressure in ambient temperatures on dry metallic powders such as Fe and Al led to a full densification by plastic deformations to achieve cold welding due to the ductility of metallic materials. In the case of inorganic compounds such as Alumina (Al_2_O_3_), no plastic deformation was observed resulting in less densification due to the repacking of grains as the applied pressure was below the yield strength of grains [13]. Other low temperature sintering techniques also exist for the fabrication of biocomposites such as hydrothermal sintering. Hydrothermal reaction can be defined as “any heterogenous chemical reaction in the presence of a solvent (whether aqueous or non-aqueous) above room temperature and at pressure greater than 1 atm in a closed system” [14]. Sintering is achieved through thermal treatment of the green bodies or powders creating grain bonding into a predominantly solid structure via mass transport events occurring on the atomic scale. The bonding by formation of necks leads to improved strength and a lower system energy. The mechanisms for sintering depend on the conditions within the system, whether there is liquid present as well as solid, and whether there is an additional external applied pressure. Diffusion in liquid state is much more rapid than solid state diffusion and can exist at lower temperature. Bouville and Studart [15] demonstrated that cold isostatic pressure (CIP) on powder in wet conditions of powder for ceramics (CaCO_3_) leads to sintering in presence of water at room temperature of an inorganic compound moderately soluble in water. Ion dissolution and liquid induced plasticity is not favored either in oil or in air, however, a dissolution-precipitation and/or plastic deformation caused by solvent (water in this case) could lead to the densification of the powder [15]. When the grains are in contact under external force, there is a high stress concentration induced by pressure solution creep caused during the movement of ions. Dissolution of the contact point takes place in the high stress region in the presence of the liquid interface layer. The dissoluted particles precipitate in the less stressed zone. This dissolution at the grain contact point is akin to dissolution-precipitation processes that are observed in the cold sintering process and in the hydrothermal sintering process [16]. Plastic deformation will also be favored in the metallic powders in addition to the dissolution-precipitation process leading to consolidation under high pressure in presence of a liquid medium. In the case of the ceramic powders, the dissolution and precipitation process plays a major role in consolidation in the presence of high pressure and a liquid interface layer. The role of liquid is indispensable for consolidation with high pressure and low temperatures. 

**Figure 1:**
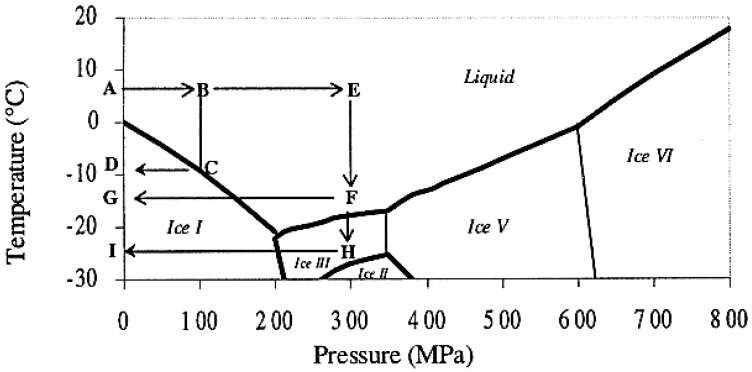
Le Bail [18]—Possibilities of high pressure processing based on the phase diagram of water.

In nature, only the crystals of ice I exist; pressure sintering of ice crystals is relevant for the densification of glaciers. The necks form between grains of ice crystals by diffusion and plastic flow due to the pressure [17]. In the phase diagram of water, it can be inferred that there is a possibility to retain the liquid form of ice even at −20 °C in the presence of high pressure (around 200 MPa). The pressure-temperature diagram of water is already well discussed by many authors in the literature [18]. From the phase diagram of water, the possibility to variate the formation and shape of ice crystals and to control the size by different ways by selecting the coupled pressure/temperature were already explored in pressure-assisted freezing and pressure-assisted thawing applied in foods [18]. The control of formation of ice crystals favors fine nucleation without growing or, in contrast, to permit the growth of a massive number of small ice crystals and also to obtain large crystals of ice I, ice III, or Ice V regarding the level of pressure, as shown in the Figure 1, adapted from Le Bail et al. [18]. Ice VI exists at ambient temperature under very high pressure, higher than 600 MPa.

Figure 1 shows various pathways possible for the formation of ice crystals:

a/Freezing in liquid phase

Water is in liquid state up to −21 °C under 210 MPa; this state could be the starting state for crystallization of ice crystals. This liquid phase could be reached by different pathways and could be like an initial state for the processes described below.

ABEF: freezing without ice crystal formation, water stays in liquid phase
b/Phase transition under constant pressure
  - ABCD: pressure-assisted freezing (PAF), freezing under a constant pressure.
  - DCBA: pressure-assisted thawing (PAT), thawing under a constant pressure, occurring within short time.
c/Phase transition by pressure change

Pressure is easier to modify than temperature, which is subjected to the mass inertia of the pressure-vessel. The pressure shift on the frozen mass allows smaller and more homogeneous ice crystals during the starting of level of pressure from depressurization at constant temperature, and also the rate of depressurization.

ABEFG: pressure shift freezing (PSF), freezing process increases the ice-nucleation rate.
d/Phase transition under a pressure change continued at a constant pressure.
GFEBA: pressure-induced thawing (PIT), reverses process of PSF.
e/Phase transition under a pressure change continued at a constant pressure.
ABEFHI: freezing to ice III and IHFEBA: thawing to ice III, ice III forms with higher density than liquid water and ice I.

Crystallization and liquefaction are controlled by level of pressure and temperature, but also by the pressurization and depressurization rate which could be very rapid; the temperature rate is more complicated to control quickly in a pressured vessel.

In the present work, we have worked on using ice as a template similar to a porogen and apply the pressure at the same time to consolidate the materials. The possibility to freeze the water at different temperatures and to create different morphologies of ice in the presence of high pressure is explored. This innovative technique called freeze isostatic pressure (FIP) consists on the application of pressure at minus temperature on a mixture of sample made of powder and water. Water is used as a template under solid state which is eliminated after return to ambient condition of pressure and temperature. Powder made of inorganic compound is compacted on such template which gives the porosity [19]. FIP allows the control of the ice crystals nucleation/growing of water. Water is easily eliminated by return at ambient conditions through sublimation. The objective by using the parameters *P* and *T*, is to select the path for selecting the crystals I, III, and V. The characteristics of such ice are as follows: Ice I (0.92 g·cm^−3^), Ice III (1.14 g·cm^−3^), and Ice V (1.23 g·cm^−3^). Both ice III and V are denser than ice I and liquid phase. The rate of compression and decompression, and freezing and thawing, determine the final size of porosities initiated by crystallization of ice crystals. At the contact point between grains of powder inorganic compound, the dissolution and precipitation into the interfacial water film occurs and could be also activated by a solid-state welding processes (cold-welding) process to obtain a well consolidated monolith. The dissolution localized at grains contact favored by high stress contact point acts like the dissolution and precipitation processes expected for HyS process, explained previously where temperature enhances this phenomenon. The transportation of species along the grain boundary in the less stress contact point leads to a precipitation on the grain surface and initiates the neck formation in the free spaces between grains, acting like osmotic pressure effect.

**Figure 2:**
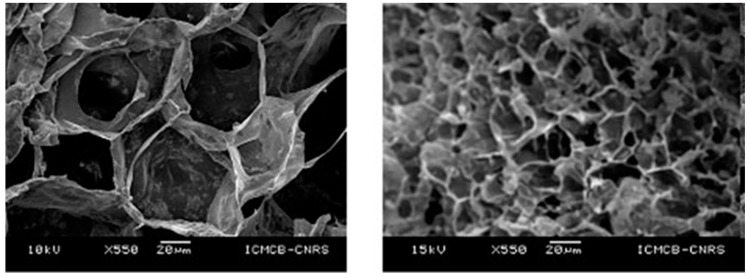
Porous TiO_2_ ceramics obtained by FIP processing.

Then, we could assume that hydrothermal reaction as “any heterogenous chemical reaction in the presence of a solvent (whether aqueous or no aqueous) under liquid state at pressure greater than 1 atm in a closed system” because the solvent under liquid phase—even if temperature is negative—could enhance dissolution and precipitation by pressure at the contact point of the grains. This consolidation between grains could be also enhanced by adding binder such as collagen, gelatin, polymer, and so on. Figure 2 shows the TiO_2_ anatase porous ceramics obtained by applying various pressures and temperatures. An example of pressure and temperature condition which yielded porous TiO_2_ ceramics was the pathway FG following a return to ambient temperature with the extraction of the sample from the pressure vessel. Interconnected pores were obtained at the aforesaid experimental conditions.

## 4. Materials and Methods

**Figure 3:**
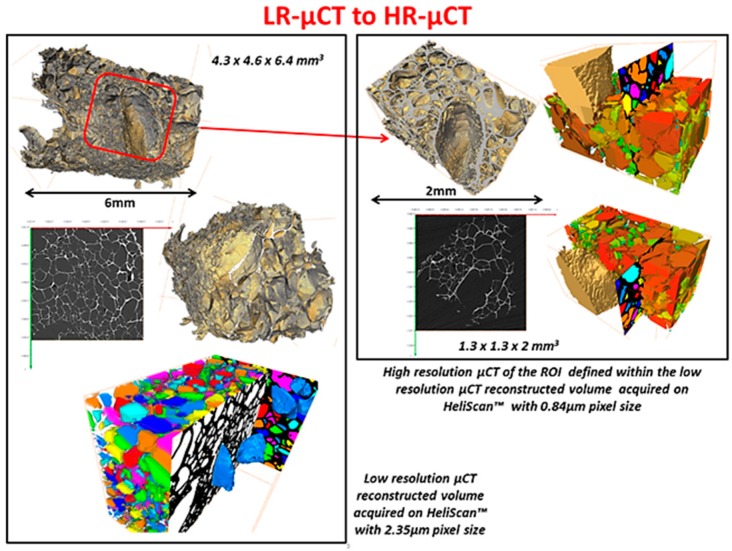
Correlating low resolution (LR) µCT to high resolution (HR) µCT. ROI = region of interest.

**Figure 4:**
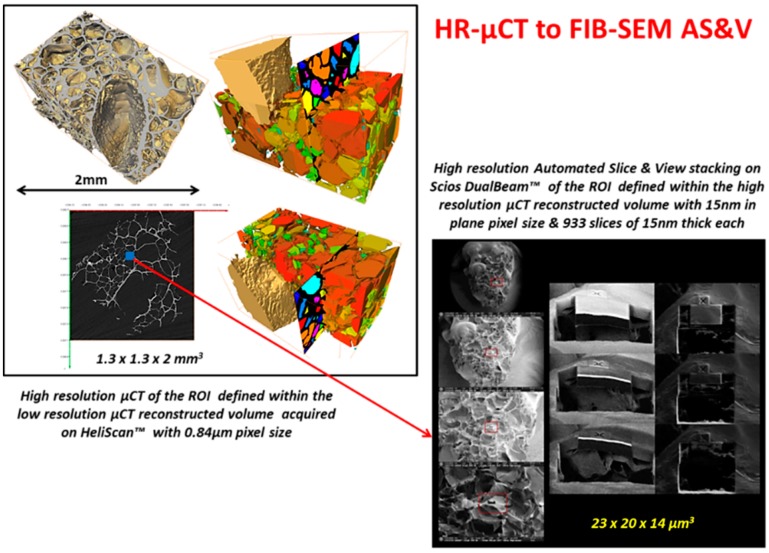
Correlating high resolution µCT to FIB-SEM Stacking.

**Figure 5:**
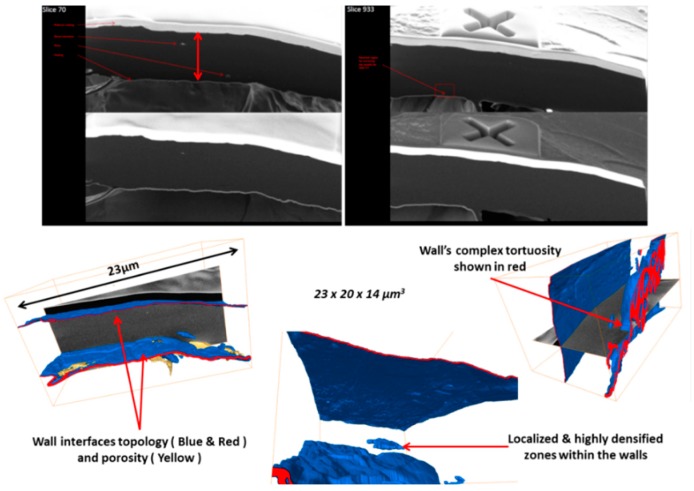
Example of porous wall’s thickness variation, topology, and tortuosity.

**Figure 6:**
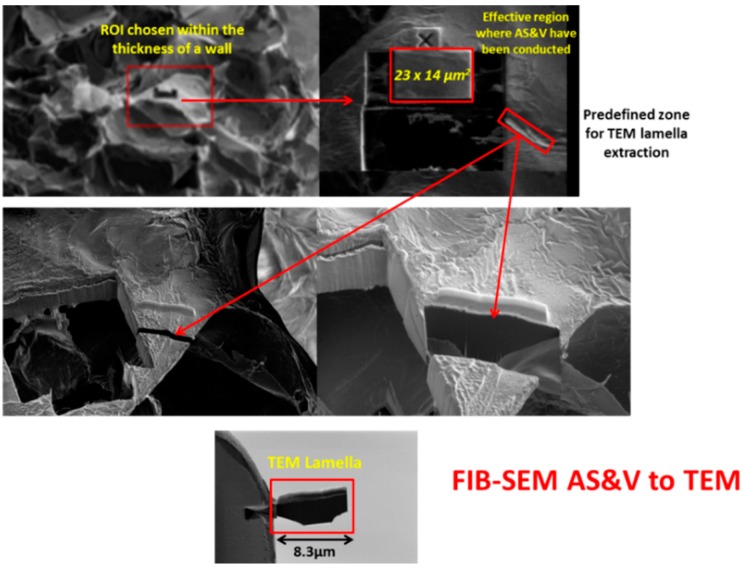
Defining and extracting ROI for TEM lamella.

**Figure 7:**
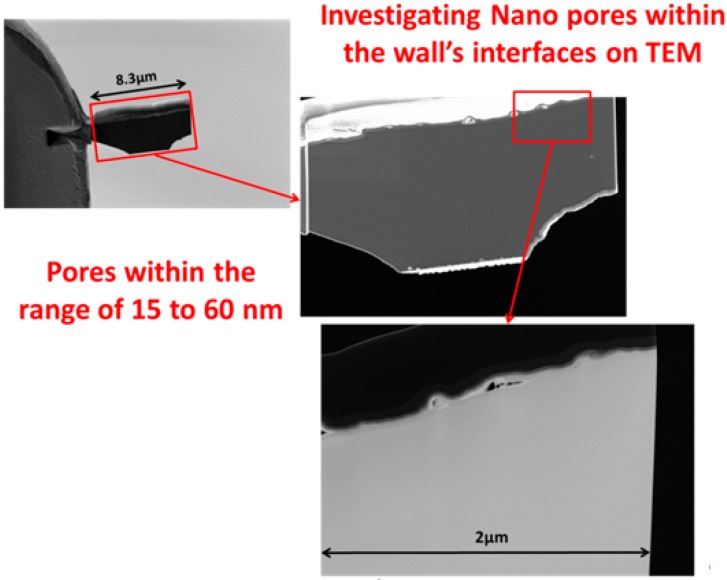
TEM Lamella correlation.

**Figure 8:**
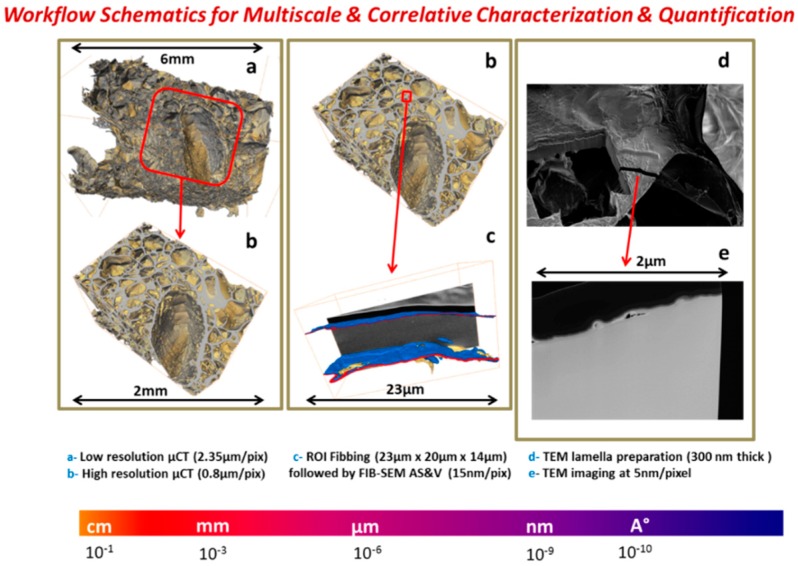
Schematics of full correlation workflow.

As noted above, an innovative fabrication methodology of the porous monolith was presented in this article. ICMCB, France has developed a very low temperature processing method that could help in obtaining high strength materials with the help of parameters such as pressure, temperature, and dwell time. The aforesaid method of consolidation yielded the monolith with porosity in the range of 80%–93%. We have used Titanium oxide (TiO_2_) in anatase phase (grain size ~15 nm). The porous monolith consisted of high porous body with fine thin pore walls and interconnected spaces. The presence of the interconnected pores will assist in cell proliferation. With the currently existing imaging techniques, it is difficult to differentiate the thin pore walls in the materials fabricated at low temperatures with little mechanical strength. Therefore, the aim of the current study was to demonstrate the feasibility and the necessity of a multiscale and multi-modal correlative approach to the characterization and structural quantification of such a material system as detailed below and shown in Figure 3, Figure 4, Figure 5, Figure 6 and Figure 7. Figure 8 shows a summarized and schematic correlative workflow used for this study.

Low resolution µCT on HeliScan™

- The biocomposite sample with dimensions of 4.3 mm × 4.6 mm × 6.4 mm was examined to select smaller region of interest (ROI) for further high-resolution imaging. The sample was scanned with double-helix trajectory with projections per revolution set to 2880. Total amount of the acquired projections was 10,180. The volume had an isotropic voxel size of 2.35 μm. AvizoTM software was applied for building 3D model of the sample and selection of the ROI for further investigation. Sample block of 1.3 mm × 1.3 mm × 2 mm was cut off using a scalpel according to the 3D model based defined ROI.

High resolution µCT on HeliScan™

- The sample ROI was examined afterwards, using HeliScan™ system. The voxel size of the reconstructed images was 0.84 μm.

High resolution Automated Slice & View stacking on Scios DualBeam™ microscope

- Simultaneous slicing with FIB and SEM imaging was performed on FEI’s Scios DualBeam™ microscope (Merignac, France). Auto Slice and View 4.0 software was used to automatically collect 3D data from a user-defined volume of the sample by milling serial sections (slices) and then acquiring high resolution images of each slice. This software enabled study of the 3D structure and composition of samples at the nanometer scale.
- Area of interest of 23 µm × 20 µm × 14 µm was analyzed, thickness of a unique slice removed by FIB being 15 nm, total of 933 slices were milled. SEM imaging was done with SE and BSE in-lens detectors simultaneously, image resolution set to 1536 × 1024 pixels. In this way, voxel size is 16 nm × 20 nm × 15 nm.

High resolution TEM

- A TEM thin foil sample was lifted out near the previously done Slice and View site. Due to the delicate nature of the sample, the lifted out chunk was thinned to 300 nm thickness with 30 kV ion beam. 5 kV and 2 kV ion beam cleaning was then performed to make the sample electron transparent.

**Figure 9:**
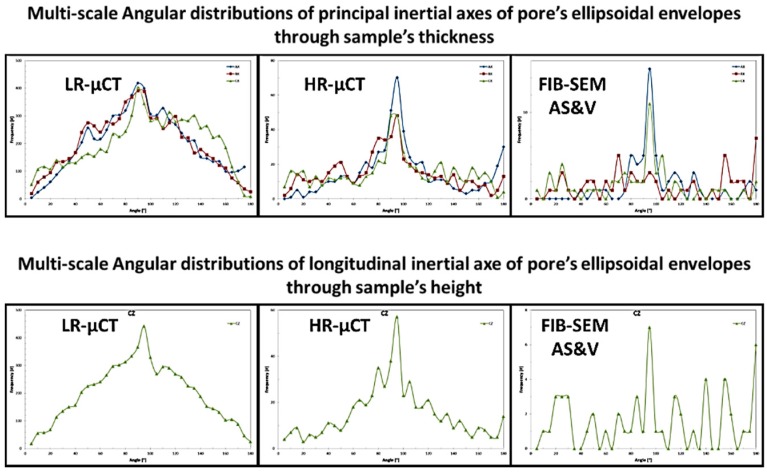
Porous media quantification throughout scales.

**Figure 10:**
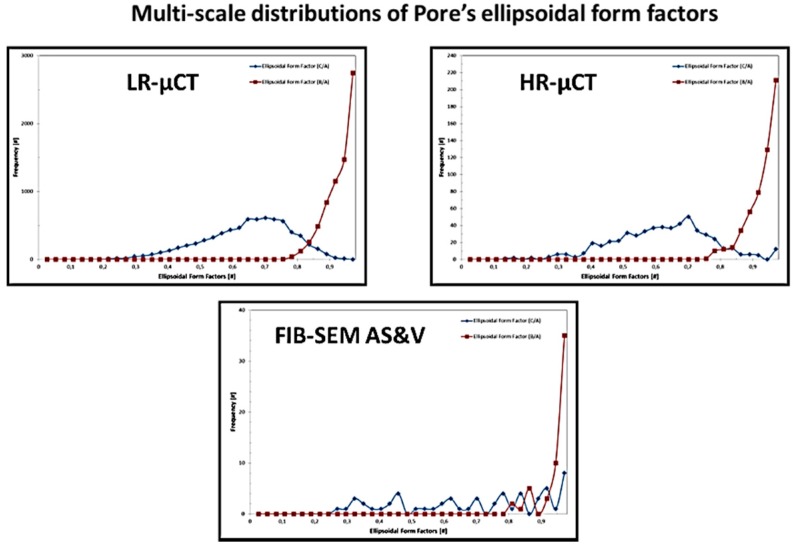
Porous media quantification throughout scales.

**Figure 11:**
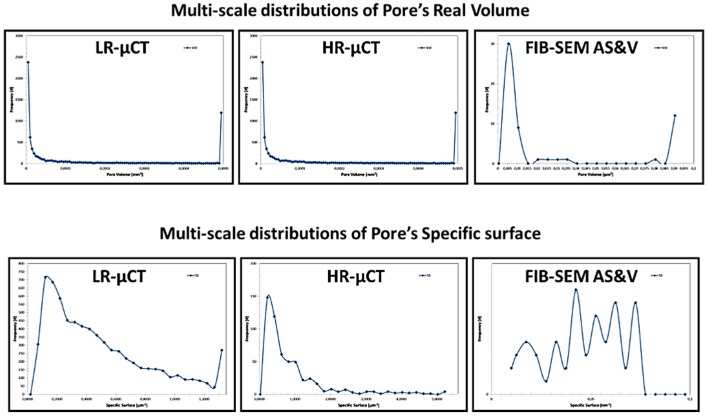
Porous media quantification throughout scales.

**Figure 12:**
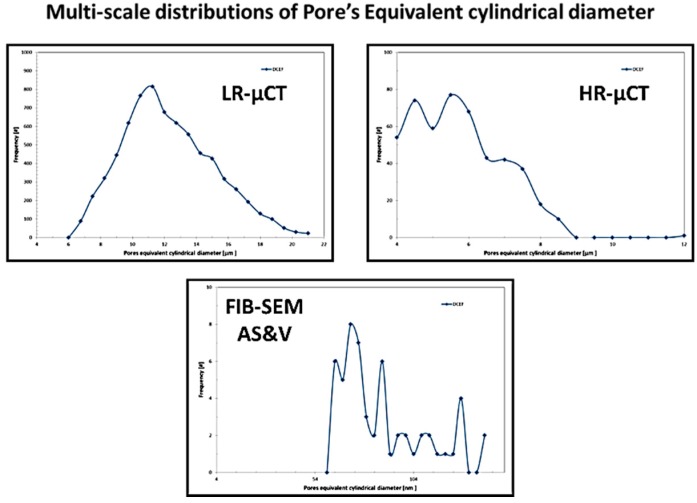
Porous media quantification throughout scales.

Figure 9, Figure 10, Figure 11 and Figure 12, show quite clearly the scale overlap between porosities characterized and quantified at different scales, where the interval of size and volume variations makes multiscale correlation mandatory. Without such an approach, the complete characterization and understanding of the interconnected porous media is impossible.

Porous media morphological changes, from more elongated to purely spherical pores, while decreasing in pore size, is also observed and quantified as shown in Figure 9, Figure 10, Figure 11 and Figure 12.

To summarize quantifications, the three-dimensional interconnectivity of the porous media through different scales for the innovative manufactured biomaterial was investigated using newly developed 3D correlative and multi-modal imaging techniques.

- Multiscale X-ray tomography, FIB-SEM Slice & View stacking and high-resolution STEM-EDS electronic tomography observations were combined allowing quantification of morphological and geometrical spatial distributions of the multiscale porous network through length scales spanning from hundreds of microns to nanometer.
- 3D macro-micro-nano pore and defect network connectivity, size distribution, anisotropy, and form factors were quantified.
- 3D wall thickness distribution and localized internal wall’s densification were investigated.

## 5. Conclusions

Hydrothermal reaction could be defined as “any heterogenous chemical reaction in the presence of a solvent (whether aqueous or no aqueous) under liquid state at pressure greater than 1 atm in a closed system” because the solvent under liquid phase even if temperature is negative (like −20 °C/200 MPa for water) could enhance dissolution and precipitation by pressure at the contact point of the grains. 

The innovative process freeze isostatic pressure (FIP) based on this hydrothermal phenomenon allows the reaching of consolidated monoliths at negative temperature by using a clean template, such as water.

To validate the fabrication process, microstructural studies were mandatory and the interconnectivity of the pores. Microstructural studies carried out in the present work enabled the quantifying and qualifying of the scaffolds for tissue engineering. To identify the interconnectivity further microstructural analyses and software were developed. Three-dimensional-correlative and multimodal imaging techniques and the software developed by Thermofisher Bordeaux was highly helpful in quantifying the highly porous biomaterial for tissue engineering applications. Hence, it is foreseen that both the fabrication process and the 3D–correlative and multimodal imaging alongside the software developed can be successfully employed for other porous materials for energy or biomedical applications.
